# Dexamethasone blunts postspinal hypotension in geriatric patients undergoing orthopedic surgery: a double blind, placebo-controlled study

**DOI:** 10.1186/s12871-021-01232-w

**Published:** 2021-01-11

**Authors:** Tarek M. Ashoor, Noha S. Hussien, Sherif G. Anis, Ibrahim M. Esmat

**Affiliations:** 1 Department of Anesthesia and Intensive Care, Ain Shams University, Cairo, Egypt; 2 Department of Anesthesia and Intensive Care, Faculty of Medicin, Ain Shamse University, Cairo, Egypt

**Keywords:** Anesthesia, subarachnoid, Geriatric, Hemodynamics, Dexamethasone

## Abstract

**Background:**

Post-spinal anesthesia (PSA) hypotension in elderly patients is challenging. Correction of PSA hypotension by fluids either colloids or crystalloids or by vasoconstrictors pose the risk of volume overload or compromising cardiac conditions. Dexamethasone is used to treat conditions manifested by decrease of peripheral vascular resistance. The research team was the first to test the hypothesis of its role in preventing or decreasing the incidence of PSA hypotension.

**Methods:**

One hundred ten patients, aged 60 years or more were recruited to receive a single preoperative dose of dexamethasone 8 mg IVI in 100 ml normal saline (D group) (55 patients) 2 h preoperatively, and 55 patients were given placebo (C group) in a randomized, double-blind trial. Variations in blood pressure and heart rate in addition to the needs of ephedrine and/or atropine following spinal anesthesia (SA) were recorded. SA was achieved using subarachnoid injection of 3 ml hyperbaric bupivacaine 0.5%.

**Results:**

Demographic data and the quality of sensory and motor block were comparable between groups. At 5th, 10th minutes post SA; systolic, diastolic and mean arterial pressures were statistically significant higher in D group. At 20th minutes post SA; the obtained blood pressure readings and heart rate changes didn’t show any statistically significance between groups. The need for ephedrine and side effects were statistically significant lower in D group than C group.

**Conclusion:**

Post-spinal anesthesia hypotension, nausea, vomiting and shivering in elderly patients were less common after receiving a single preoperative dose of dexamethasone 8 mg IVI than control.

**Registration number:**

ClinicalTrials.gov Identifier: NCT 03664037, Registered 17 September 2018 - Retrospectively registered, http://www.ClinicalTrial.gov

## Background

Spinal anesthesia (SA) is preferred by anesthetists in elderly patients however, its common and sometimes dangerous complications may limit its use. Hypotension and bradycardia are the most frequent complications reaching up to one- third in non-obstetric populations [1]. The main cause of post spinal anesthesia (PSA) hypotension is the decrease in the sympathetic outflow causing arterial vasodilatation, a decrease in venous return and consequently the activation of the Bezold Jarish reflex (BJR) [2] that elicits a triad of bradycardia, vasodilatation and further hypotension [3, 4]. BJR is elicited by activation of 5-HT_3_ receptors within the intracardiac vagal nerve endings [5]. Those effects are prominent in geriatric patients with PSA hypotension estimated to be over 70% [5]. On the other hand, methods that are used to avoid the PSA hypotension (e.g., volume loading or vasopressor administration) may add the risk of hypervolemia and/or myocardial ischemia for those patients [6].

Dexamethasone (DEX) – a synthetic glucocorticoid- is used to abolish PSA nausea and vomiting [7] or PSA shivering [8], and may increase the duration of sensory block [9, 10]. DEX increases tissue peripheral vascular resistance (PVR) in rats [11] and humans by a variety of mechanisms [12] and there is a plethora of studies confirming its role in maintaining the integrity of circulation in situations of intense vasodilatation like septic shock [13] and anaphylaxis [14]. Moreover, glucocorticoids in general inhibit 5-HT_3_ expression and DEX was found to decrease the level of 5-HT_3_ in the cerebral cortex and hippocampus in developing rats [15].

This study was conducted to evaluate the efficacy of a single preoperative dose of DEX 8 mg intravenous infusion (IVI) to attenuate the PSA hypotension in geriatric patients undergoing orthopedic surgery.

## Methods

The study protocol was approved by the institute ethics committee (FMASU R 46/ 2018) before enrollment of patients and a written informed consent was obtained by every patient. This randomized, prospective, double blind, study, adheres to CONSORT guidelines and was conducted at Ain-Shams University Hospitals from the 1st of March 2018 till the 31st of August 2018 on 110 ASA I, II, III patients aged 60 years or more and scheduled for lower limb orthopedic surgeries under SA. Patients with contraindication to SA (e.g., coagulopathy, thrombocytopenia, allergy to local anesthetic agent) and those on steroids or serotonin related medications (e.g., selective serotonin reuptake inhibitor) were excluded.

Randomization of the patients was performed using a computer-generated random numbers concealed in sealed opaque envelopes and a nurse randomly chose the envelope that determined the group of assignment. Patients were allocated into two equal groups (55 each) with 1:1 ratio according to post spinal hypotension prophylaxis; patients received either DEX 8 mg diluted in 100 ml 0.9% normal saline (NS) IVI over 15 min (D group) or an equal volume of plain NS (Control group) (C group) 2 h preoperatively. Study medications were prepared by the hospital pharmacy and given by anesthetists not involved in any other part of the study. Patients fasted 8 h and were not intravenously hydrated before the procedure.

On arrival in the operating room, routine monitoring was applied, a venous access was established with a wide-bored cannula and patients were sedated by 1 mg of midazolam IV. Infusion of NS solution was commenced not to exceed 400 mL during SA and for 20 min thereafter. SA was performed with the patient in the setting position by injecting 3 ml of 0.5% hyperbaric bupivacaine solution (Marcaine® Spinal Heavy 0.5%; Sunny pivacaine, Manufactured by Sunny Pharmaceutical - Cairo - Egypt) at L3-L4 or L4-L5 level using 25gauge Quincke spinal needle.

After completing the subarachnoid injection, patients were positioned supine. Sensory level (by alcohol swab) and motor block (by Modified Bromage Score [16]) were assessed every 5 min. Heart rate (HR), systolic blood pressure (SBP), diastolic blood pressure (DBP) and mean blood pressure (MBP) were recorded before giving the study medications (base line) and then during SA every 5 min for 4 readings.

Hypotension was considered if there was 25% decrease below the baseline for MAP and was managed by 300 ml of NS solution with incremental intravenous 5 mg doses of ephedrine. The proportion of patients with hypotension at any time during the first 20 min after induction of the SA and before starting the surgical procedure was considered as the primary outcome of the study.

Bradycardia was considered if the heart rate was less than 50 beats/min and was treated with atropine (0.01 mg/kg) IV. Only data before giving ephedrine and/or atropine were analyzed if they were given. The anesthetists who performed the spinal anesthesia and recorded the hemodynamic changes were unaware of the medications given to patients preoperatively.

The research team considered all 4 measurements of each of 3 blood pressures (SBP, DBP&MAP) as secondary outcomes. The obtained blood pressure values at each study time point were compared to the minimal values recorded within the 20 min following the subarachnoid blockade. Surgical procedure, positioning of the patient or application of tourniquet was not allowed during the study period. The pattern of sensory and motor blocks, number of patients who needed atropine and/or ephedrine, changes in HR among groups and associated side effects (e.g., nausea, vomiting and shivering) were considered as secondary outcomes.

### Statistical analysis

Depending on Baig et al. [17], who found that the hypotension rate in ondansetron and normal saline groups were 7.5 and 28.3% respectively and assuming that the power = 0.80 and α = 0.05 and by using PASS 11th release, the minimal sample size for an equal size controlled clinical trial was 50 in each group. We recruited 55 in each group for possible attrition [18].

The collected data were coded, tabulated, and statistically analyzed using IBM SPSS statistics (Statistical Package for Social Sciences) software version 18.0, IBM Corp., Chicago, USA, 2009.

Descriptive statistics were done for quantitative data as minimum& maximum of the range as well as mean (SD) (standard deviation) for quantitative normally distributed data, while it was done for qualitative data as number and percentage.

Inferential analyses were done for quantitative variables using Shapiro-Wilk test for normality testing, independent t-test in cases of two independent groups and paired t-test in cases of paired data. In qualitative data, inferential analyses for independent variables were done using Chi square test for differences between proportions and Fisher’s Exact test for variables with small expected numbers. The level of significance was taken at *P* value < 0.050 was significant, otherwise was non-significant.

## Results

**Fig. 1:**
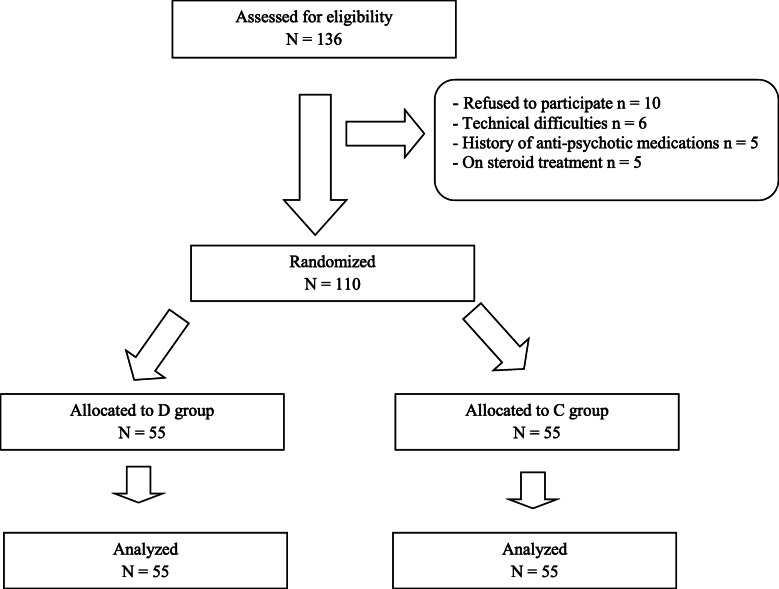
Flow chart of the study

Out of 136 patients were assessed for eligibility and 110 patients were analyzed; 55 in the D group and 55 in C group **(**Fig. 1**).** Demographic data in the study population were comparable between groups with no statistically significant differences between them (Table 1).

**Table 1:** Demographic and baseline clinical characteristics among the studied cases

Variables	D Group (***n*** = 55)	C Group (***n*** = 55)	***P***-value
Age; years	75.8 (5.4)	75.6 (5.0)	^0.826
Sex; male/female	22/33	25/30	#0.563
Weight; kg	67.7 (3.8)	66.8 (3.2)	^0.217
Height; cm	167.3 (4.3)	166.4 (4.9)	^0.316
ASA; I, II, III; n	4/ 38/ 13	7/ 33/ 15	#0.519
Ischemic heart disease (IHD); n	33	39	#0.229
Diabetes Mellitus (DM); n	20	21	#0.844
Renal impairment; n	11	10	#0.808
Hypertension (HTN); n	42	45	#0.482
Anti hypertensive medications; n	42	45	#0.482
• Beta-Blockers	36	40	#0.409
• ACE	29	32	#0.565
• ARBs	7	8	#0.781
• CCB	6	5	#0.751
Valvular heart diseases; n	15	12	#0.506
• Aortic stenosis (mild)	5	4	*§*0.999
• Aortic stenosis (moderate)	4	3	*§*0.999
• Mitral regurgitation (moderate)	6	5	#0.751
Surgical procedure			
• Total hip	13	15	*§*0.839
• Total Knee	14	12	
• Hip hemi-arthroplasty	17	14	
• DHS	11	14	

Angiotensin II receptor blockers (ARBs), Dynamic Hip Screw (DHS)

^Independent t-test

#Chi square test

§Fisher’s Exact test

Assessment was done after subarachnoid block and ended 20 min later. Number of segments above S1 was not statistically significantly lower in C group than in D group at 5th minute and afterwards. Number of segments above S1 significantly increased in both groups beginning from 5th minute and afterwards. Time to complete motor block was not statistically significantly shorter in D group than in C group (Table 2).

**Table 2:** Sensory and motor blocks among the studied cases

Variables	D Group (***n*** = 55)	C Group (***n*** = 55)	Difference(D-C)		^***P***-value (groups)	#***P***-value (versus minute-5)
			Mean (SE)	95% CI		
**Number of segments above S1**						
5th minute	4.0 (0.8)	3.8 (0.7)	0.2 (0.2)	−0.1–0.5	0.174	–
10th minute	5.5 (0.8)	5.2 (1.0)	0.3 (0.2)	0.0–0.7	0.056	< 0.001*
15th minute	6.7 (0.9)	6.4 (0.9)	0.2 (0.2)	0.0–0.6	0.167	< 0.001*
20th minute	7.0 (0.8)	6.7 (0.6)	0.2 (0.1)	0.0–0.5	0.097	< 0.001*
**Time to complete motor block**						
**Time (minutes)**	7.1 (0.8)	7.4 (0.9)	−0.3 (0.2)	−0.6–0.1	0.126	–

^Independent t-test

#Paired t-test

*Significant

**Fig. 2:**
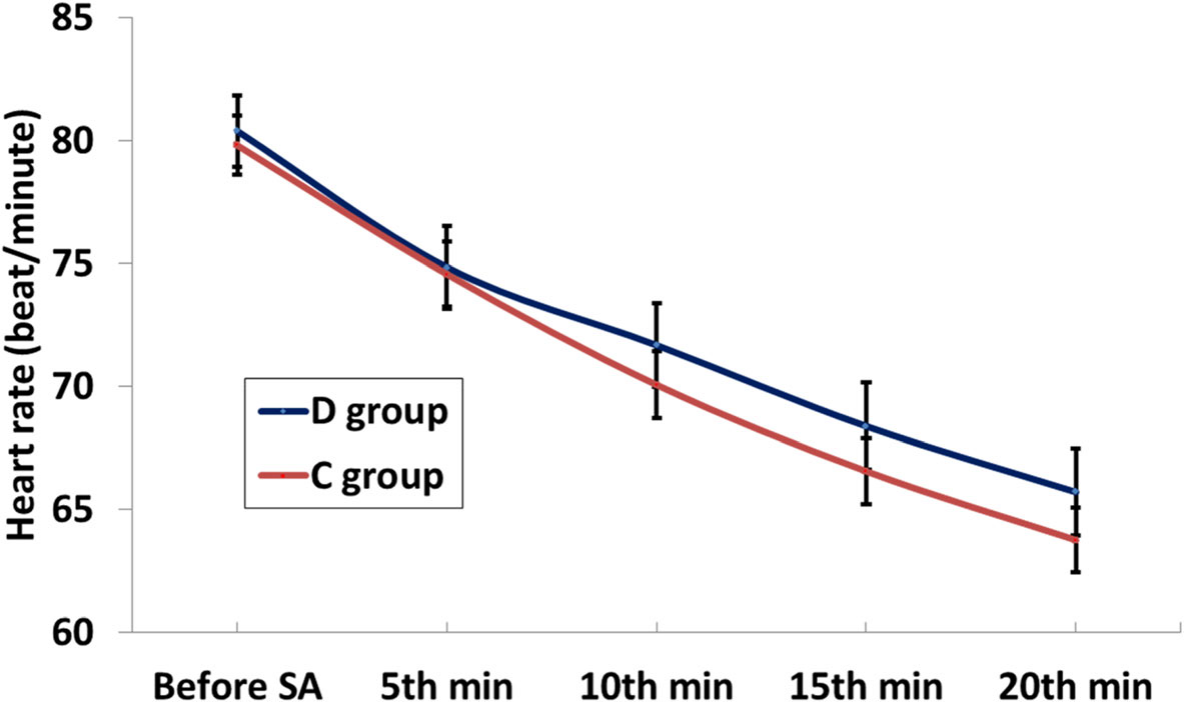
Changes in HR among groups

SBP at 5th and 10th minutes were statistically significantly higher among D group than C group, while DBP and MBP at 5th,10th, and 15th minutes were statistically significantly higher among D group than C group (Table 3). There weren’t statistically significant changes between groups in HR (Fig. 2).

**Table 3:** Blood pressure changes among the studied cases

Variable	D Group (***n*** = 55)	C Group (***n*** = 55)	Difference (D-C)		^***P***-value (groups)	#***P***-value (versus baseline)
			Mean (SE)	95% CI		
**SBP (mmHg)**						
Baseline	147.9 (7.5)	147.9 (8.0)	0.1 (1.5)	−2.9–3.0	0.951	–
5th minute	142.3 (10.2)	133.7 (15.2)	8.6 (2.5)	3.7–13.5	0.001*	< 0.001*
10th minute (*N* = 52, 47)	139.8 (9.5)	134.7 (9.9)	5.1 (1.9)	1.3–9.0	0.009*	< 0.001*
15th minute (*N* = 50, 41)	137.2 (9.3)	135.0 (8.2)	2.2 (1.9)	−1.5–5.9	0.246	< 0.001*
20th minute (*N* = 48, 38)	134.1 (8.3)	132.1 (6.9)	2.0 (1.7)	−1.3–5.3	0.236	< 0.001*
Minimum	131.4 (10.6)	125.2 (13.3)	6.2 (2.3)	1.7–10.8	0.008*	< 0.001*
**DBP (mmHg)**						
Baseline	86.8 (6.1)	86.5 (6.6)	0.4 (1.2)	−2.0–2.8	0.753	–
5th minute	77.8 (7.5)	72.6 (9.2)	5.1 (1.6)	2.0–8.3	0.002*	< 0.001*
10th minute (*N* = 52, 47)	75.4 (6.7)	70.5 (7.6)	4.9 (1.4)	2.0–7.7	0.001*	< 0.001*
15th minute (*N* = 50, 41)	72.5 (7.7)	67.8 (8.1)	4.7 (1.7)	1.4–8.0	0.006*	< 0.001*
20th minute (*N* = 48, 38)	67.8 (6.9)	65.7 (7.1)	2.1 (1.5)	−0.9–5.1	0.174	< 0.001*
Minimum	66.6 (7.3)	63.0 (7.9)	3.6 (1.5)	0.7–6.5	0.015*	< 0.001*
**MBP (mmHg)**						
Baseline	107.2 (6.5)	106.9 (7.0)	0.3 (1.3)	−2.3–2.8	0.826	–
5th minute	99.3 (8.3)	93.0 (11.1)	6.3 (1.9)	2.6–10.0	0.001*	< 0.001*
10th minute (*N* = 52, 47)	96.9 (7.4)	91.9 (8.2)	5.0 (1.6)	1.8–8.1	0.002*	< 0.001*
15th minute (*N* = 50, 41)	94.0 (8.1)	90.2 (8.0)	3.8 (1.7)	0.5–7.2	0.026*	< 0.001*
20th minute (*N* = 48, 38)	89.9 (7.0)	87.8 (6.9)	2.1 (1.5)	−1.0–5.1	0.178	< 0.001*
Minimum	88.2 (8.0)	83.7 (9.2)	4.5 (1.6)	1.2–7.7	0.008*	< 0.001*

^Independent t-test

#Paired t-test

*Significant

**Fig. 3:**
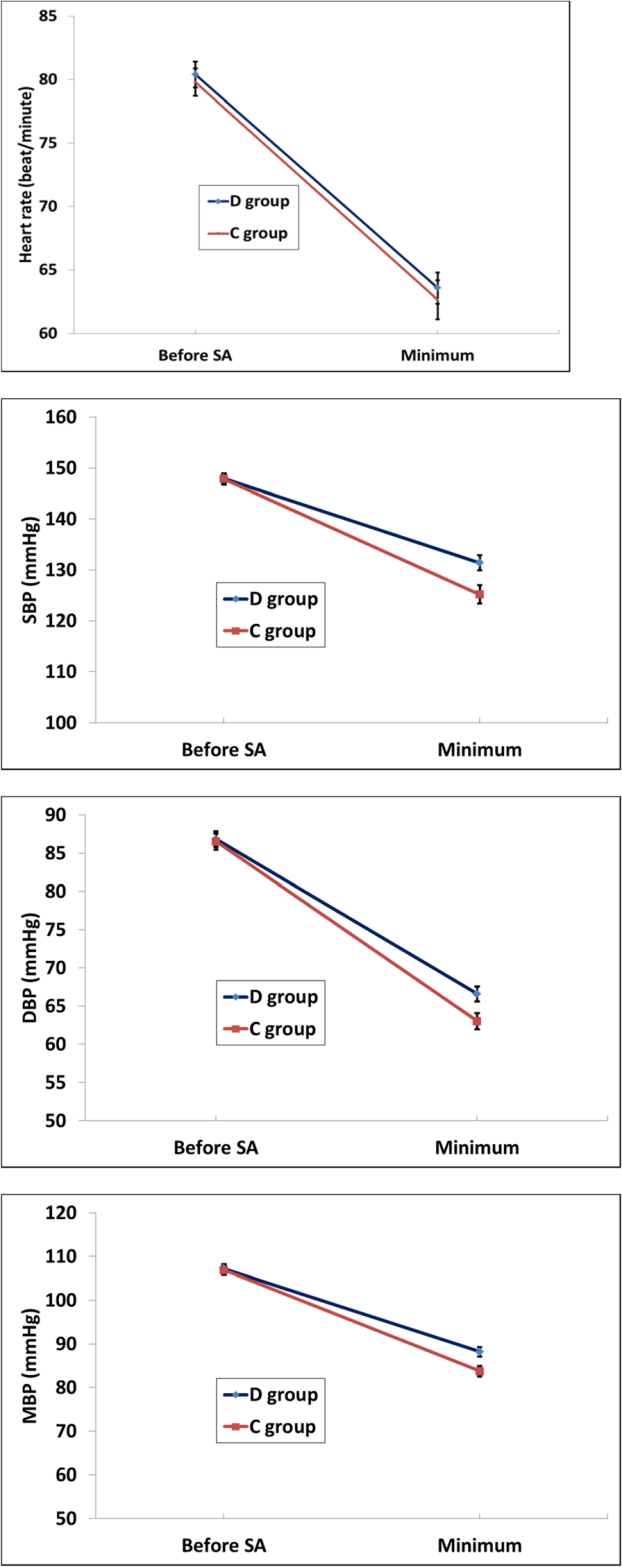
**a** Minimum HR, **b** Minimum SBP, **c** Minimum DBP, **d** Minimum MBP changes between groups

Minimum readings in SBP, DBP and MBP were statistically significantly higher in D group than C group, while the minimum reading in HR showed no statistically significant differences between groups (Fig. 3).

The need of ephedrine was statistically significantly lower in D group (*P* = 0.025) than C group, while there were no statistically significant changes in the need of atropine (*P* = 0.303) or those who needed atropine and ephedrine (*P* = 0.429) between D and C groups. The time to ephedrine and/or atropine need was not statistically significant between groups. Nausea, vomiting and shivering were statistically significantly lower in D group than C group (*P* = 0.012, *P* = 0.032, *P* < 0.001 respectively) (Table 4).

**Table 4:** Atropine and ephedrine needs and associated side effects among groups

Variables	D Group (***n*** = 55)	C Group (***n*** = 55)	***P***-value	RR (95% CI)
**Primary outcome**				
Patients developing PSA hypotension; n, %	8 (14.5%)	18 (32.7%)	#0.025*	0.44 (0.21–0.94)
**Hemodynamic supports**				
Atropine; n,%	7 (12.7%)	11 (20.0%)	#0.303	0.64 (0.27–1.52)
Ephedrine; n,%	8 (14.5%)	18 (32.7%)	#0.025*	0.44 (0.21–0.94)
Atropine and Ephedrine; n,%	7 (12.7%)	10 (18.2%)	#0.429	0.70 (0.29–1.71)
Time needed for atropine (minute), (*n* = 7,11)	8.6 (3.8)	7.7 (3.4)	^0.631	
Time needed for ephedrine (minute), (*n* = 8,18)	10.6 (5.6)	9.2 (4.6)	^0.493	
**Associated side effects**				
Nausea; n,%	7 (12.7%)	18 (32.7%)	#0.012*	0.39 (0.18–0.86)
Vomiting; n,%	1 (1.8%)	8 (14.5%)	§0.032*	0.13 (0.02–0.97)
Shivering; n,%	7 (12.7%)	25 (45.5%)	# < 0.001*	0.28 (0.13–0.59)

## Discussion

This study demonstrated favorable response rates regarding the efficacy of a single preoperative dose of DEX 8 mg intravenous infusion (IVI) to attenuate the PSA hypotension in geriatric patients undergoing orthopedic surgery. The research team observed higher minimal values of systolic, diastolic and mean arterial pressures in DEX group with minimal effects on heart rate. As far as the authors know, they were the first to raise that observation and proposed that theory.

The investigators observed that patients who were on steroids for different reasons and had spinal anesthesia had favorable post-spinal hemodynamic outcomes with minimal hypotension and accordingly minimal needs for vasoconstrictors. This proposed the theory of the value of administration of DEX for obtunding the PSA hypotension.

Different controlled trials failed to demonstrate the superiority of either general or neuroaxial anesthesia on the outcome in elderly patients. However, neuroaxial anesthesia benefits of minimizing surgical stress, reducing pulmonary compromise, superior pain control and reduction of total blood loss made it a well-accepted anesthetic option for geriatric patients [19]. However, in elderly, SA is associated with 25–69% incidence of hypotension and decreased physiological reserve that if added to the associated cardiovascular ischemic and/or valvular disease makes even brief episodes of uncorrected hypotension hardly tolerable and might cause detrimental consequences on their cardiac and mental compromised conditions [20].

**Fig. 4:**
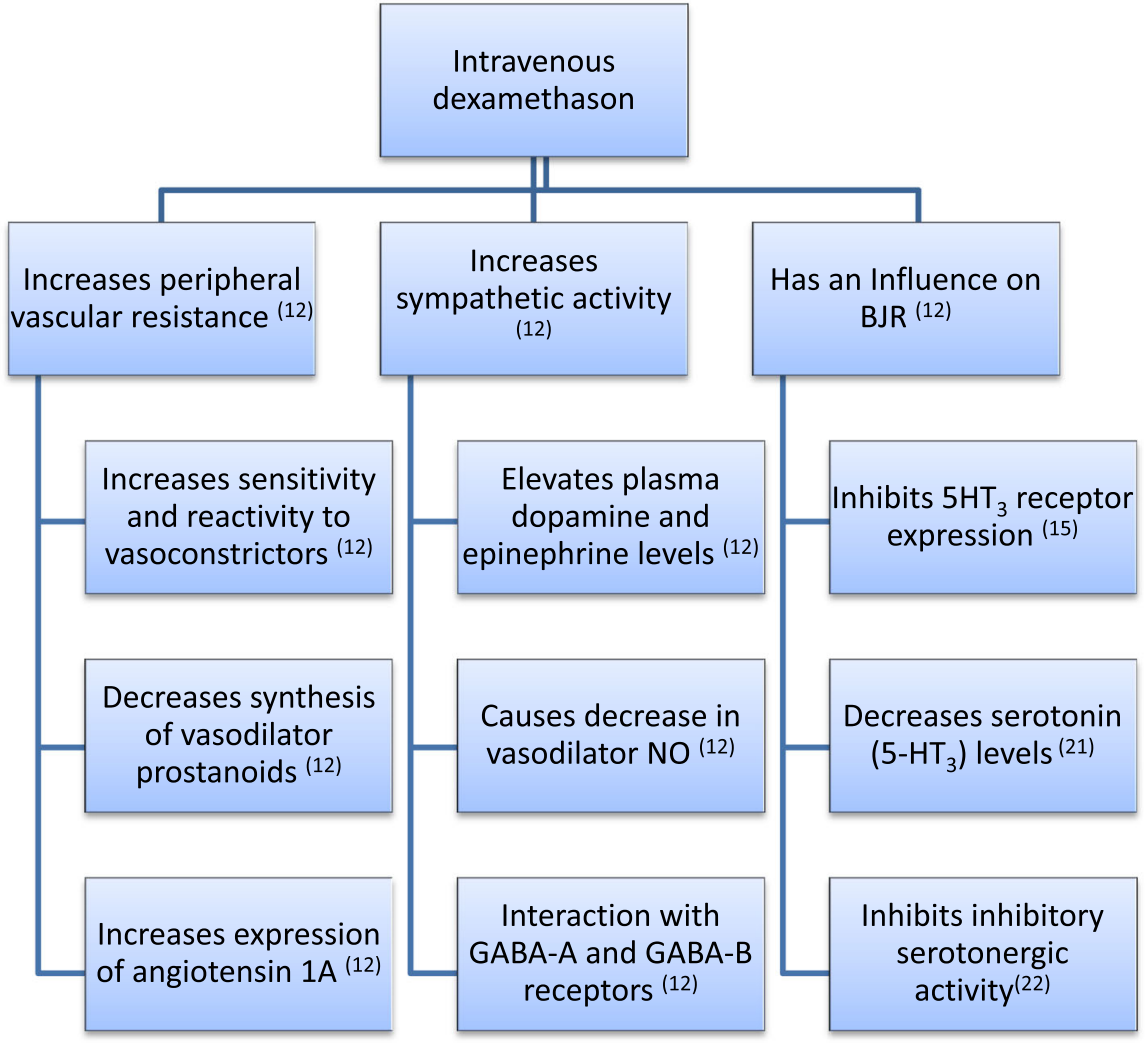
Possible mechanisms of Dexamethasone in alleviating spinal anesthesia induced hypotension

DEX is a potent synthetic glucocorticoid that has pure glucocorticoid activity [12]. It increases PVR by a variety of mechanisms i.e., decreases vasodilator nitric oxide (NO), increases sympathetic activity and elevates plasma dopamine and plasma epinephrine. It also increases the sensitivity of vascular endothelium to different vasoconstrictors [12]. Moreover, it has an anti 5HT_3_ effects which might influence BJR [12]. Those two effects hit exactly the two pathophysiological effects incriminated in eliciting post spinal hypotension [2], that explained our results and confirmed our conclusion (Fig. 4) [12, 15, 21, 22]. The timing and the administration of a preoperative single dose of dexamethasone IVI was based on a meta-analysis study conducted by De Oliveira et al. [7]. ,The investigators believed that all patients were given the same dose of hyperbaric bupivacaine since all patients were given 3 ml of hyperbaric bupivacaine without additives.

Methods to alleviate post spinal hypotension either physical e.g., leg wrapping, elastic stockings, optimizing patient’s position, or pharmacological e.g., intravenous fluids and vasopressors have been used with varying degree of success [23]. The usual measures of pre-load or co-load of either crystalloid or colloid remain controversial with many studies that confirmed that post-spinal hypotension remains significant regardless of the type or timing of the given fluids [23] and may cause hypervolemia [6]. Infusion of crystalloid solutions results in its redistribution to extravascular compartment and induces atrial natriuretic peptide secretion which might augment lowering blood pressure because of its natriuretic, diuretic, and vasodilatory effects [24]. Infusion of colloid solutions on the other hand, despite remaining in intravascular space for a longer duration, is not popular routinely due to its increased cost, possibility of derangement of coagulation, suppression of platelet activity and the risk of anaphylaxis [25]. When it comes to vasoconstrictors, they cause tachycardia and hypertension which may worsen associated myocardial ischemia [26].

The study of Owczuk et al., documented that administration of intravenous ondansetron (5HT_3_ receptor blocker) prior to spinal anesthesia in geriatric patients attenuated the drop in the diastolic and mean arterial pressure without substantially affecting the systolic blood pressure [5]. However, meta-analysis studies fail to confirm the validity of those conclusions based on low quality of evidence and insufficient evidence [27]. Moreover, ondansetron might be responsible for lower spinal block level and early recovery from spinal anesthesia [28].

In concordant with our study, Chu et al. [15], confirmed that dexamethasone (with 5-HT_3_ receptor blocking properties) similarly reduced the PONV risks as has been shown with other 5-HT_3_ receptor antagonists e.g. ondansetron. Concomitant with our results, Moeen et al. [8], reported that intrathecal dexamethasone was as effective as intrathecal meperidine in attenuation of PSA shivering compared to placebo in patients scheduled for prostate surgery under spinal anesthesia with less adverse events. Adding to this, Shalu et al., concluded that administration of DEX 8 mg IV prolonged the duration of sensory block and postoperative analgesia in patients undergoing lower segment cesarean section under spinal anesthesia [9].

This study had some limitation namely; the relatively small sample size and the restriction of the study duration to 20 min post spinal. However, the investigators chose to restrict the time of study to the time of maximum hemodynamic instability and before other influences e.g., tourniquet, skin incision or, bleeding occur. The time frame of 20 min may be considered as inadequate for a drug such as corticosteroids, which acts with a transcriptional mechanism on nuclear receptors, and therefore takes several hours to reach the peak of action. However, this could be argued by the fact that dexamethasone was given 2 h before commencement of spinal anesthesia and the onset of its action is within 10 min for the IV route [29]. Glycemic profile in the hours after dexamethasone administration, the rate of infection and the postoperative delirium should have been reported.

On the other hand the small narrow differences may be considered as a limitation. However, the statistical significance was emphasized by the clinical significance of obtunding post-spinal hypotension without the need for infusing volume or the use of hemodynamic support e.g., ephedrine. The investigators even observed that patients who did develop hypotension needed lower doses of ephedrine and hypotension when developed was not associated with nausea and/or vomiting. The research team strongly suggests trying the use of dexamethasone in geriatric patients and other patient populations with higher risk of postspinal hypotension like obstetric patients. Moreover, ephedrine, by virtue of its synthetic origin, may rarely cause allergic reactions, such as contact allergic responses with topical use (e.g., during ophthalmologic surgery) [30], and delayed severe dermatitis following IV injection has been reported [31].

The study however had many merits; the most important of which was the redirection of the medical society towards using dexamethasone which offers a cheap, an available and a simple pharmacological strategy to prevent post-spinal hypotension, a common complication of spinal anesthesia, which can lead to serious events. Moreover, dexamethasone can be used also for the prevention of postoperative nausea and vomiting, the prevention of post spinal shivering and as antalgic adjuvant.

In conclusion, despite the small differences and a short time frame assessment in the study, the investigators found that the single administration of 8 mg dexamethasone IVI prior to spinal anesthesia in geriatric patients attenuated the decrease in arterial blood pressure, especially in whom excess fluid infusion or alpha-agonist administration is contraindicated due to the risk of cardiovascular decompensation.

## Conclusion

A single preoperative dose of dexamethasone 8 mg IVI attenuated the postspinal hypotension in geriatric patients undergoing orthopedic surgeries.

## Data Availability

The data that support the findings of this study are available from Ain-Shams University Hospitals and they were not publicly available. Data were however available from the authors upon reasonable request and with permission of Ain-Shams University.

## Abbreviations

SA: Spinal anesthesia
PSA: Postspinal anesthesia
BJR: Bezold Jarish reflex
5HT_3_: 5-hydroxytryptamine receptor type 3
DEX: Dexamethasone
PVR: Peripheral vascular resistance
ASA: American Society of Anesthesiologists
SNOSE: Ratio in opaque and sealed envelope
NSS: Normal saline
I.V.: Intravenous
HR: Heart rate
SBP: Systolic blood pressure
DBP: Diastolic blood pressure
MBP: Mean blood pressure
SD: Standard deviation
NO: Nitric oxide
